# Clinical Applications of Neuropsychoanalysis: Hypotheses Toward an Integrative Model

**DOI:** 10.3389/fpsyg.2021.718372

**Published:** 2021-09-09

**Authors:** Daniela Flores Mosri

**Affiliations:** Department of Psychology, Psychoanalytic Psychotherapy, Neuropsychoanalysis, Universidad Intercontinental, Mexico City, Mexico

**Keywords:** neuropsychoanalysis, clinical applications, affective neuroscience, consciousness, psychoanalytic treatment

## Abstract

Neuropsychoanalysis has been established as a field based on the dialog between psychoanalysis and the neurosciences. Freud was a neurologist for 20 years and used the neuroscientific knowledge of his time as the foundation of his metapsychology. Psychoanalysis has predominantly relied on its own method to develop techniques for the different psychoanalytic treatments. It rarely uses contributions from fields outside psychoanalysis that could enrich its understanding of the mind. Neuropsychoanalysis has informed and revised several topics in psychoanalysis, for example consciousness and the unconscious, dreams, and affect amongst many others. Clear clinical applications of neuropsychoanalysis can be appreciated in the work with neurological patients. However, a constant question from clinicians is whether neuropsychoanalytic findings can contribute to psychoanalytic treatments with non-neurological patients. This paper explores clinical applications of neuropsychoanalysis mainly based on affective neuroscience to propose an analysis of emotions that may contribute to the gradual development of a neuropsychoanalytically informed psychotherapy. The task of integrating neuroscientific knowledge into psychoanalytic technique is still considered a challenge of accentuated complexity, but it is at the same time a necessary and promising endeavor that aims at improving the quality of the treatments available for human suffering and psychopathology.

## Introduction

Neuropsychoanalysis has been around for over 20 years. Clinically speaking it began its applications by offering psychoanalytic psychotherapy to neurological patients (Kaplan-Solms and Solms, 2000). The benefits of this approach have filled an important gap in the therapeutic work with these patients because it acknowledges that there is a subjective experience that needs to be addressed as part of the treatment. However, it has taken longer to use the knowledge of the dialog between psychoanalysis and the neurosciences in the clinical work with non-neurological patients. It is a consensus amongst clinicians interested in neuropsychoanalysis to affirm that their work has been modified when they integrate neuroscientific findings to their practice. However, few papers seem to directly address the topic (e.g., Johnson, 2009, 2010; Lane et al., 2015; Flores Mosri, 2017; Solms, 2018b). The latter does not seem fortuitous. Integrating neuroscientific findings with psychoanalytic theory has proved to be a challenging task. How necessary is it to undertake such an endeavor? This paper intends to describe the reasons why it is beneficial for clinical practice to work from a neuropsychoanalytically informed approach. The document covers some of the main topics in neuropsychoanalysis that may have clinical relevance for the psychotherapeutic practice with non-neurological patients, followed by the proposal of a technique mainly based on affective neuroscience that clinicians may find useful to complement the already valuable psychoanalytic method. A dialectical way of conducting clinical work from neuropsychoanalysis is gradually becoming a neuropsychoanalytically oriented psychotherapy.

## The Challenges of Psychoanalytic Treatments

Over more than a hundred years of psychoanalytic practice, its technique has been helpful to a number of people who have looked for a relief of symptoms in the consulting room. It has been renowned as a psychotherapeutic approach that has long-term effectiveness as it attempts to comprehend how symptoms originate based on unconscious processes. It is generally accepted that going back in the past to look for the origins of a symptom is useful and distinctive of psychoanalytic technique. However, its own strengths can result equally problematic, e.g., the reconstruction of memories takes time, usually years which may be too long for some symptoms. A consequence of the long-term process that psychoanalysis implies is its high cost which may render it inaccessible to some people. Another drawback is found in its unpredictability in terms of the duration and potential outcome.

Other relevant issues can be found within the psychoanalytic theory and technique. One of them is the existence of many schools in psychoanalysis that tend to fight against one another instead of attempting an integration of their claims. On the one hand, having different perspectives within psychoanalysis can constitute its unique richness. On the other, too many schools may end up splitting clinical knowledge that could be at the cost of the clinician’s performance in the consulting room. But perhaps the most important implication is that it makes it hard for a person looking for help to know which approach would be best for them. Furthermore, the results of psychoanalytic treatments can vary widely from one clinician to the other. That is a natural constituent of a subjective process, yet it is unclear if the technique is what works or if it depends on the person applying it.

An added difficulty for people in search of treatment can be to know which psychotherapeutic approach is best for them, i.e., the distinction between psychoanalysis and psychodynamically or psychoanalytically oriented psychotherapies, whose differences can be hard to discriminate even for some clinicians in the field. Further problems will be detailed in other sections of this paper, but what is clear is that psychoanalysis could benefit from the dialog with other fields of knowledge to work on its weaker features (Kandel, 1999; Govrin, 2019).

The next question to address is why there should be a dialog with the neurosciences. Freud was a neurologist for 20 years before he created psychoanalysis (Sacks, 1998) which means that the basic science of psychoanalysis is neuroscience (Johnson and Flores Mosri, 2016) and that there would be no psychoanalysis at all without Freud’s neurological work (Flores Mosri, 2019b). Taking account of the well-known correlates of the brain and the mind, it is no wonder that the neurosciences are crucial for Freud’s proposal of a metapsychology (Solms, 2021). Furthermore, when one topic is investigated from one viewpoint only, it is more susceptible of bias and of partial comprehension. Psychoanalysis tends to trust excessively in its own method (Solms and Turnbull, 2002). Seldom does it look for additional findings coming from other fields of knowledge. A constant concern is whether it can still be defined as psychoanalysis if it opens to a dialog with other fields of knowledge. Neuropsychoanalysis in its definition follows a dialectical approach (Solms and Turnbull, 2011). Hence, a neuropsychoanalytically informed clinical practice should result advantageous when it can consider the valuable contributions of two perspectives. The best known neuropsychoanalytical model for clinical work is Mark Solms’ clinical implications workshop, which he has given in several countries and that is currently available online. From there, the International Neuropsychoanalysis Society started a clinical register. There is also a paper by Solms (2018b) that addresses clinical issues. His ideas will be discussed as a foundation for further clinical efforts to work from a dialectical viewpoint, including the integrative method here proposed.

## Neuropsychoanalaytically Informed Topics in Clinical Work

Neuropsychoanalysis has made contributions on several topics, including consciousness and the unconscious, memory, dreams, defense, sexuality, and drives amongst others. But probably the theme that has interested clinicians the most is affect. Panksepp’s affective neuroscience (Panksepp, 1998; Panksepp and Biven, 2012) and his proposal of seven basic emotion systems has been the starting point to address the need to reassess clinical work. Take for example the statement of a conscious id (Solms and Panksepp, 2012; Solms, 2013) which could hardly go without clinical implications. To build an integrative approach, three main steps are highlighted. (1) To identify and acknowledge paradoxes and ambiguities in psychoanalytic concepts. This constitutes a relevant issue as clinical work is based on hypothetical concepts. If a concept is obscure or entails a theoretical paradox, then its application to clinical work is just as vague or imprecise as the concept. (2) To integrate knowledge from different fields and perspectives into psychoanalytic theory can contribute to inform psychoanalytic topics and potentially help solve some of the conceptual and clinical problems described before. (3) To benefit from the already existent integrative attempts in neuropsychoanalysis, both theoretical and clinical. As simple as it may seem, once neuropsychoanalytic knowledge has been developed, the next challenge is to know how to apply it in the clinical situation. The following sections will cover some of the potential modifications that may come when clinical work integrates neuropsychoanalytic knowledge.

### The Conceptual Problems in Psychoanalysis

The problem of conceptualizing in psychoanalysis starts with Freud’s writings. His work was ongoing, and it was not infrequent that he changed his views about certain topics and hence, needed to modify the concepts in his theoretical work. Sometimes he explained the reasons for these changes and informed readers about new data integrated into previous ideas. At other times he did not clarify that a concept had been modified or the reasons to use a different perspective. The latter has given Freud as many interpretations of his work as readers are which is hardly a problem if it only relates to a theory of the mind. However, if this theory is the foundation for clinical work, more accuracy would be beneficial. An associated outstanding difficulty has been the imprecisions of the translation of Freud’s papers and books. A new edition of Freud’s complete psychological works (Solms, 2018a) may contribute to clarifying some of the conceptual issues related to drives and instincts just to mention one example. The unreliability on the various editions has led to confusion and misleading interpretations of Freud’s work.

To summarize, Freud’s theory of the mind is the pilar of psychoanalytic practice and still the most comprehensive. As mentioned before, if it holds paradoxes and obscure concepts, clinical work becomes uncertain as well. Neuropsychoanalysis has contributed to the revision of several of the most problematic topics, e.g., the unconscious quality of the id (Solms and Panksepp, 2012; Solms, 2013), repression (Boag, 2012; Kessler et al., 2017; Solms, 2018b), and recently Solms’ revision of the Oedipus complex (in Flores Mosri, 2020; Solms, in press b) and drives (Solms, in press a). When a definition remains essentially the same throughout an author’s work its main characteristics stand out and refine the concept. In contrast, when the description changes several times throughout the author’s work, it becomes ambiguous. Additional to the fact that a good theory needs harmony in its concepts (Balint, 1968), if that theory is used to guide clinical work it is then crucial to elucidate its potential paradoxes. One noteworthy example was the topic of a whole congress of the International Neuropsychoanalysis Society, i.e., repression (to read about the diverse perspectives addressed in this event, see Flores Mosri, 2018). One of the goals of psychoanalytic therapies is to overcome repression, namely, to turn unconscious contents of the mind into conscious ones. However, if repression can be understood in different ways that imply different meanings, then one of the most important clinical goals of psychoanalysis is unclear.

Another crucial problem with concepts in psychoanalysis relates to the many schools in which two main tendencies can be identified. The first is that different schools use the same concept for different phenomena. The concept of “structure” for instance, has been used to signify different things by different authors (e.g., Freud, 1923, 1938; Fairbairn, 1954; Bergeret, 1974). The second tendency associates with different schools and authors using different concepts or names for equivalent phenomena; minor variations seem to necessitate a different name or concept without acknowledging other authors’ contributions to the same topic. Disagreement amongst clinicians can be naturally expected, nevertheless, when the essential elements are abstracted, the main sense tends to overlap importantly. The immense advantage of such overlap is the possibility of confirming one hypothesis across different schools and authors in psychoanalysis. Yet, the consequent problem is that few authors attempt an integration of overall psychoanalytic knowledge. Hence, the lack of potential discussions and integration of knowledge may be a loss for psychoanalysis in general and particularly for contributions to clinical work. Moreover, a dialog with other fields could be facilitated by clear conceptual foundations. For instance, if the concept of repression has different meanings for different authors and clinicians, attempting to operationalize it for further research renders almost impossible (e.g., see Boag, 2006; Erdelyi, 2006). How clinicians conceptualize repression may change the whole objective of their work. And repression is only one example amongst many. Psychoanalytic theory can benefit from detailed revisions to facilitate more testing of concepts (Kandel, 1999) and an ongoing clarification of how the mind works that in turn may promote more quality in clinical approaches. To that end, Govrin (2006) used Ryle’s concept of “thick descriptions” to convey the need for complex theories of the mind in psychoanalysis. By expressing his concern about not generating new knowledge in psychoanalysis, Govrin highlights the need for the creation of new grand theories of the mind that may explain empirical problems that bring about hypotheses to be tested. The first step hence is the recognition of those problems that have a direct impact on clinical work.

### The Unconscious Mind, Repression, and Memory

Despite the different schools and perspectives in psychoanalytic thinking the foremost topic remains the unconscious mind. The idea that making the unconscious conscious is the main goal of psychoanalytic treatment may be challenged by neuroscientific findings or at least its sense may change after considering some important facts. The dynamic unconscious is based on the notion that repression turns excessively painful experiences into unconscious split representations. Freud’s first proposal referred to biographical episodes that were forgotten due to the intolerable affect that accompanied them (Breuer and Freud, 1893-1895; Freud, 1894, 1895/1950). The mental process that explains this amnesic state is defense which has the objective of weakening the intensity of a negative affect^1^. Nevertheless, the use of a defense implies a compromise formation that gives rise to symptoms. Unconscious contents attempt to recover their original conscious quality. After 1897, Freud (in Boag, 2006) modified his original conceptualization of the repressed and stated that what becomes unconscious is one of the parts that triggers conflict between opposing tendencies, i.e., drives and/or desires. The symptom would then be a symbol or a metaphor of the repressed and would resolve when the unconscious content reached consciousness. If it is accepted that a painful conscious experience can become unconscious and that its cost is symptom formation, it then makes sense to aim at making the unconscious conscious. However, the knowledge that there are several memory systems (Milner et al., 1998) was not available to Freud. Alberini (in Flores Mosri, 2020) has asserted that Freud encountered the problem of having to explain multiple psychological processes relying on only one memory system. What we now know is that an experience is coded in multiple memory systems which renders it hard to forget, at least in its entirety. Furthermore, memory responds to Ribot’s law which claims that older memories are less vulnerable to forgetting which can be explained by consolidation processes (Alberini, 2005). Hence, the idea of a memory that is fully forgotten is unlikely. Mnemonic diversity allows for a memory to be stored and to have an influence on behavior (Solms and Turnbull, 2002). However, there is a number of clinicians that seem to stick to the idea of forgotten repressed autobiographical memories. Boag (2006) considered this fact as a pathology of science with enormous implications on how clinical work is conducted. Neuropsychoanalytic contributions also explain how forgetting a traumatic episode may not relate with the notion of repression.

The diverse memory systems recruit different anatomical regions and circuits. If one memory system is impaired, the other systems may remain functional (Milner et al., 1998) as extensive studies with patient HM demonstrated^2^ (Squire, 2009). Psychoanalytic technique relies predominantly on declarative memory, namely, episodes that can be described in words. If an experience cannot be expressed verbally, it is generally thought that it is repressed. The latter can be challenged when the different memory systems and the way in which they work are considered. Memories can fail to be declared if the episodic memory system that relies on the hippocampus is not involved in memory formation. One known cause relates to stressful experiences that activate the hypothalamic-pituitary-adrenal axis (HPA axis). If a person goes through a traumatic event, it may be the case that the release of cortisol blocks the function of the hippocampus (LeDoux, 1996; Yovell et al., 2015) which in turn impedes the formation of an episodic memory of the event. Thus, the episode is not forgotten or repressed; it was simply not coded in an episodic format in which it could be retrieved verbally. Yet, the experience will have an influence on the person’s behavior due to its codification as an emotional memory that recruits the amygdala. Fear conditioning is an accurate form of memory which associates a particular context with an emotional state; this kind of association cannot be deleted as it is meant to predict future similar situations. The person is then able to avoid dangerous contexts to protect their life and integrity in the future.

Implicit memory has been linked to the unconscious (Kandel, 1999). However, associative learning can be conscious or unconscious under different conditions. The distinction depends on whether associative learning recruits the hippocampus or not. Many associations are learned implicitly and thus, cannot become declarative. The latter is important to understand unconscious contents. As a classical imaginary example, if Pavlov’s dog visited a psychoanalyst’s office to try to stop salivating when it hears bells, the dog would not be able to tell the psychoanalyst about the conditions in which the association was established. Furthermore, even if the dog could put the whole episode into words, that would still not modify the association (see Alberini’s opinion in Flores Mosri, 2020). People are constantly exposed to spontaneous associative learning that is not coded episodically. This type of unconscious memory does not relate to repression and the dynamic unconscious in its traditional conceptualization. Notwithstanding it plays a crucial role on mental life. A neuropsychoanalytic approach to this type of formulation can clarify that some unconscious contents are not susceptible of becoming cognitively conscious. Given this condition, it seems relevant to question how useful it would be to put them into words and how else they can be tackled during psychoanalytic treatments.

Hence, clinical work needs access to those procedural and emotional memories through different methods. Freud (1938) suggested that transference was an essential part of psychoanalytic treatment. Therefore, understanding the different types of memories is relevant for clinical work, e.g., short-term working memory and long-term explicit and implicit memory systems. It has been hypothesized that transference relates to procedural and emotional memory (Turnbull et al., 2006; Moore et al., 2017). Knowing how these systems work can help to conduct more precise technical interventions during the treatment. More about the implications of this type of unconscious memories will be addressed in terms of affect in other sections of this paper.

The question now turns to what it is that could be considered making the unconscious conscious and its benefits as a psychotherapeutic instrument. Several times in the consulting room a person may claim that they do not remember certain periods of their lives, frequently their childhood. If asked directly, the person may report not having access to those memories. Whether that is due to resistance or defense is not simple to discern. If asked in an indirect way, people tend to retrieve memories of the period that they claimed that was not available for recall. It is unlikely that the experience was repressed. A viable explanation is that when the ego is not alerted then the memory is recoverable. According to Marty (1990) and Solms (2013), these memories were preconscious. When Freud (1894; Breuer and Freud, 1893-1895) still referred to repression as a motivated forgetting of painful episodes, he claimed that the defensive process began with the voluntary decision to keep the episode at a distance from consciousness. Nonetheless, if the essence of unconscious contents is their unknowable quality, then they cannot be made conscious. Representations can be considered preconscious and not unconscious; some of them are more available to be retrieved according to their given affective value. Representations linked to positive affect would be more available while images associated to negative affects may be less accessible. However, traumatic representations can be too available against the person’s will, e.g., in posttraumatic stress disorder (LeDoux, 1996) which adds complexity to the topic of conscious and preconscious contents. And how useful is it therapeutically speaking to retrieve preconscious experiences? Breuer and Freud (1893-1895) thought that the discharge of tension was the solution to neurotic symptoms. The discharge could happen either through movement or through association. Speaking about traumatic events led to movement and thus discharge. Abreaction or catharsis would then represent the beginning of the talking cure. Freud elaborated on Breuer’s initial model when he realized that verbalizing experiences that were characterized by negative affect was not sufficient to solve symptoms. He discovered the transference and proposed its analysis (Freud, 1909, 1938) and later described countertransference. Yet, the ultimate psychoanalytic goal was facilitated when working through was achieved (Freud, 1914). Freud’s psychotherapeutic method then encompassed a series of steps toward the cure that included verbalizing and comprehending mental contents, identifying the characteristics of the transference-countertransference relationship, and working through defense and repetition compulsion. It is probably working through that constitutes the most obscure concept (Laplanche and Pontalis, 1973) of the psychoanalytic method. It may be the case that a person talks about traumatic contents, understands the origins of repetitive behaviors, even those seen in the transference-countertransference relationship and that they still repeat the same symptomatic pattern. Some argue that working through is missing, but it turns challenging to achieve a final step that is not clearly defined.

## Clinical Neuropsychoanalytic Applications Based on Affective Neuroscience

The previous sections addressed some of the most important problems that can be found in psychoanalytic technique. More can be added and some of them will be tackled in the framework of affective neuroscience. The intention of the following proposal is not to solve all the difficulties derived from psychoanalytic clinical work, but to contribute with clinical hypotheses to gradually improve our current available instruments through a neuropsychoanalytically informed approach.

### The Fundamental Role of Affect

Breuer and Freud (1893-1895) quickly learned that the cause of hysteria was directly linked to affect. They proposed that unbearable affects meant an increase in the amount of energy in the nervous system. The excess of quantity had to be discharged to decrease the intensity of the negative affect. The symptom formation was a result of the operation of defense mechanisms (Freud, 1894) that aimed at decreasing the amount of subjective pain. One of the tasks of the mind is thus, to avoid unpleasurable feelings. Solms (2018b, 2019, 2020, 2021) has proposed that negative affect expresses unsatisfied needs. Panksepp (1998, 2011; Panksepp and Biven, 2012) explained that there are three types of primary affects, namely sensory, homeostatic, and emotional. They are shared by all mammalian brains and aim at survival. Solms (2021) considers that the three types of affects are homeostatic as they seek to satisfy the needs that come from the body. Negative affects indicate that there is a lack of homeostasis, i.e., that a need must be met. Unpleasurable feelings drive the mind to perform work and solve problems. In contrast, positive affects signal the return to a homeostatic state that is subjectively felt as pleasure. Panksepp and Biven (2012) declared that affects are always conscious and that they are generated at the periaqueductal gray (PAG) located in the midbrain. Affects lead to pre-wired behavioral, instinctual patterns that constitute unconditioned responses designed to favor survival. These raw affective responses constitute the primary process emotions, in Panksepp’s taxonomy, SEEKING, PANIC/GRIEF, RAGE, FEAR, LUST, CARE, and PLAY^3^ (see Panksepp’s original formulation of the seven basic emotional systems in Panksepp, 1998). Each affective system is activated by stimuli common to the members of a species as they entail the learning of predicted situations experienced through numerous generations. These genetic pre-wired reaction patterns constitute an effective evolutionary mechanism to ensure that the members of a species do not have to learn from the start what their ancestors have experienced under certain circumstances. Solms (2021) has elaborated on the view that affects express unsatisfied needs that drive the mind to perform work to meet them. In his opinion, there is a predominant need at a time that will gain conscious attention. This need should be identified in the psychotherapeutic process to particularly work on it. Solms (in press b) has also asserted that the basic emotions conflict with one another and that it is the task of the mind to solve these conflicts.

Solms’ proposal to include the knowledge of Panksepp’s affective neuroscience into psychoanalytic technique expands the meaning of affects and adds complexity to the topic. Many clinicians think that expressing affects in words solves symptomatic formulations. They may refer to an unconscious quality of affects, i.e., repressed affects, that become conscious when they are put into words and next understood and associated to representations. Just like Freud, Panksepp and Biven (2012) stated that affects are always conscious and Solms (2021) has recently highlighted this conscious quality of affect. According to his proposal, affect is felt, namely, it is experienced and constitutes the fundamental quality of consciousness. Panksepp (2011) underscored the difficulties that we may encounter to recognize raw affects due to our predominant cognitive thinking. However, he emphasized that affects are experienced whether we pay cognitive attention to our feelings or not; he declared that feeling is compulsive, i.e., we cannot stop feeling which may be due to the fact that subjective experience favors survival. This acknowledgment led to further revisions of the Freudian concept of the id. Solms and Panksepp (2012; Solms, 2013) argued that the id is conscious because of the affective nature of the core brainstem regions that generate consciousness. They relied on the reports by Merker (2007) whose work with hydranencephalic children has demonstrated clear emotional reactions and motivations in the absence of the cortex. The latter argues in favor of the affective nature of consciousness and how it relates to subcortical structures that do not recruit the cortex. Panksepp (2011) suggested nested-hierarchy types of emotional organization. Primary process emotions learn from experience and become secondary affects based on conditioned responses that depend on upper limbic regions of the brain. It is only tertiary-process emotions that need the contribution of cortical regions. Tertiary process emotions are more available to be expressed verbally as opposed to primary and secondary-process emotions. Psychoanalytic technique has kept its foundations on a cortico-centric view of affect and consciousness, possibly following Freud (1920) or perhaps not even questioning whether neuroscientific data could inform the subject of consciousness and the unconscious, and lead to relevant revisions to both theory and technique. If emotions are conceptualized as cognitive, then it makes sense to emphasize the relevance of verbalizing them. When it is acknowledged that affects are felt, whether cognitive attention is given to them or not, then a substantial revision seems essential.

Communicating feelings in verbal language may entail various restrictions. Words express as accurately as possible what is being felt, yet subjective experience cannot be translated into a precise cognitive report as its nature is to be felt. Thinking of affects can be a useful instrument for psychoanalytic treatments as it will provide some information about what a person *thinks* that they feel or felt. However, the most reliable method so far to infer what someone else feels is the transference-countertransference quality of the therapeutic relationship. This method, however, involves other restrictions. The reliability on one’s opinion about what someone else feels is always limited, as one can only feel one’s own subjectivity. Inference using empathy and cognitive knowledge may get close to what someone else feels, but there is no certainty guaranteed. Thus, the decision about what emotion is predominant in a person’s life to design a psychoanalytic treatment may be biased and may differ from one clinician to another. Hence, a method that analyzes affects considering different dimensions may constitute an effective way to comprehend what affects are signaling during psychoanalytic treatments.

To assess how affects work, two aspects are generally considered. First, the spontaneous reactions to the current context, namely, the present affective feeling. Second, a more general appraisal of the overall affective responses that the person predominantly experiences in a sustained way (i.e., mood). The analysis here proposed discusses a method to help clinicians understand their impressions about another person’s affective states. According to Panksepp and Biven (2012), affect has different dimensions by which it can be assessed and that can guide clinical work. Based on his proposal, the suggested dimensions to analyze affects in clinical work are: (1) the subjective nature of affect; (2) behavioral responses, which can be instinctual or learned from experience; (3) the cognitive ideas about what is felt; (4) the neurobiological correlates of affect. A brief description of each dimension follows (see Table 1).

**TABLE 1:** The different dimensions of affect analysis.

**1. The subjective nature of affect** - Phenomenological consciousness - Inferred from the transference-countertransference relationship
**2. Behavioral responses** - Nonverbal expressions: Gestures and body language - Instinctual behavior: Primary-process emotions - Learned behavioral patterns: Secondary-process learning Associations—mainly unconscious
**3. Cognitive ideas about feelings** - Tertiary-process cognitions based on primary and secondary affects - Affective secondary- and primary-process emotions are inferable from discourse - Preconscious representations (thing- and word-presentations)
**4. Neurobiological correlates of affect** - Hypotheses

(1) The subjective essence of affect relates to the conscious nature of feelings explained before, namely, feelings are meant to be experienced in order to indicate a homeostatic imbalance that requires that the person do something to recover homeostasis. This quality of affect can be inferred from the transference-countertransference relationship. Clinicians train to build hypotheses derived from the feeling of being with a patient. However, to rely excessively on the clinician’s impressions may lead to misconceptions as every clinician is prone to their own subjective biases. This is one of the reasons why the present proposal suggests considering other dimensions of affect that can complement or challenge the clinician’s perspective.

(2) In terms of behavioral responses, the simplest form is the nonverbal expression of affect, seen mainly in facial expressions and gesticulations. The use of the analytic couch may restrict access to this sort of information; when a person speaks without direct eye contact with another person, body language is rarely used. The face-to-face format mainly used in psychodynamic psychotherapies evokes nonverbal expressions of affect which are a valuable source of information to complement other subjective impressions of the clinician.

Other behavioral sources can be relevant to add information to the analysis of affect. Classically clinicians in psychoanalysis are trained to pay little attention to the patient’s behavior and conditionings. Yet, the free associations of patients tend to describe behaviors which following Panksepp (2011) can take mainly two forms. The first one refers to the primary instinctual behavioral patterns triggered by unconditioned stimuli, that is, unconditioned responses. An example is to explore the environment to identify potential available resources to solve our needs prompted by the subjective feeling of curiosity (SEEKING behavior) (see Table 2). The second form relates to behaviors that are learned from experience, namely, conditioned associations that evolve from the original primary instinctual patterns when they are insufficient to solve all the needs encountered in a certain environment. These learned patterns recruit long-term implicit memory systems such as the procedural and emotional subsystems. Procedural patterns automatize behavioral skills and habits and keep mainly an unconscious nature (Kandel, 1999; Solms, 2017). Associations are established by recruiting affective circuits such as in fear conditioning in which an emotional reaction can be triggered by a specific stimulus whose associative context can remain unknown to the person experiencing it. An example of the latter can be seen in specific phobias in which the person identifies the stimulus that elicits the fear reaction, but the cause of apparently senseless associations often remains unknown, that is, unconscious. Similar learning is established from early relations with others that constitute relational patterns that can be later reexperienced in the transference-countertransference relationship. In sum, behaviors are driven by affects; if clinicians pay attention to the patient’s conduct, both in the consulting room and derived from the patient’s verbal reports, valuable information can become available to complement or contrast against the clinician’s subjective impressions of what the patient is feeling.

**TABLE 2:** Brief summary of subjective feelings and behaviors derived from the activity of the basic emotion systems.

**Basic emotion system**	**Subjective feeling**	**Behavior**
SEEKING	Curiosity	Exploring behavior
	Positive expectancy Feeling excited about the future Hope	Search

LUST	Sexual attraction Pleasure	Sexual activity

CARE	Pleasurable feelings when taking care of others	Support others Take care of others Protect others

PANIC/GRIEF	Separation distress Sadness Loneliness	Separation distress vocalizations Withdrawal

PLAY	Joy Happiness	Rough-and-tumble PLAY with peers

FEAR	Fear Anxiety Stress	Freezing Flight

RAGE	Anger	Fight Aggressive behaviors

(3) With regard to the cognitive ideas about feelings, as mentioned before, psychoanalytic practice has relied on the verbal expression of affects as an essential part of the treatment. The concept of alexithymia (Sifneos, 1973) stressed the importance of identifying and describing feelings in words as this function is not available to people who keep an operational life (Marty, 1990) that makes them emphatically vulnerable to psychosomatic symptoms. Hence, the verbalization of feelings became one of the clearest objectives of psychoanalytic treatments, particularly for narcissistic and borderline personalities that do not seem to align with Freud’s ideas of intrapsychic conflict. Two main premises seem to be used to understand this therapeutic goal. First, that affect can be unconscious and second, that affect needs to be associated to representations. Based on Panksepp’s findings, neuropsychoanalysis considers that affects are always conscious. Solms (2019, 2021) has argued in favor of this position using neurobiological evidence that highlights the qualitative role of the extended reticulo-thalamic activating system and the periaqueductal gray. If affects are always conscious, it becomes a necessary question to ask why so many clinicians think that they can have an unconscious quality.

**FIGURE 1:**
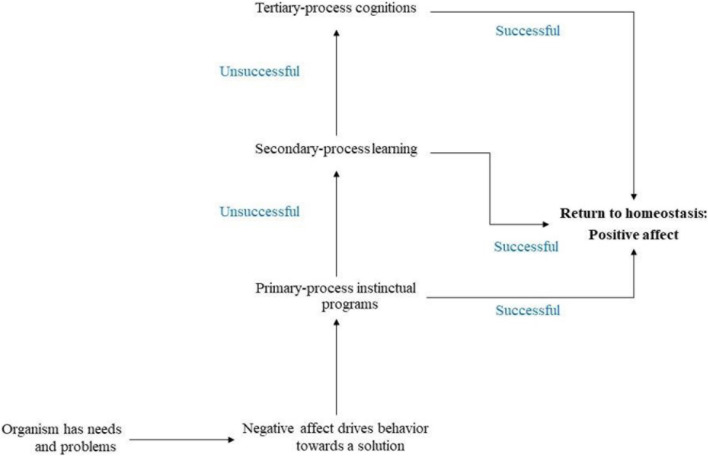
The three levels of problem solving. Figure illustrates how negative affect triggers different levels of behavior as an attempt to solve needs and problems to diminish or eliminate the subjective feeling of negative affects. When the primary-process instinctual programs fail at problem solution, the secondary-process learnings are needed. If in turn the second level is unsuccessful then the tertiary-process cognitions are required. The return to homeostasis is signaled by a positive affect.

The question can be answered at different levels. First, the various dimensions of affect lead to the need of considering its neurobiological features. To understand how affects work, there is a need to comprehend the neurobiological principles upon which they operate. Affect necessarily involves a complex neurochemistry that relies on neuroanatomical circuits, seven, according to Panksepp (1998). Affect as the indicator of somatic and other needs has at least three levels of problem-solving described in Figure 1. The level that the cognitive dimension aims at describing is that of tertiary-process cognitions. Knowing the cognitive contents of the mind entails to consider that affect is always first, i.e., every thought comes from a feeling as affect is the foundation of psychic life. Thus, when a patient describes their ideas, they are already speaking about affect and it would be the clinician’s job to infer the emotional contents from the discourse by relying on the support from the rest of the dimensions of affect analysis. It is useful to keep in mind that cognitive consciousness recruits different cortical regions including the prefrontal cortex and the short-term working memory functions to come up with a deep analysis of the problem that needs to be solved when the first two levels (i.e., the primary and secondary) have been insufficient. Thinking allows for plans of action that may be appropriate to satisfy the need that demands the cognitive conscious attention of the person. Then clinicians can be sure that the contents of a patient’s thoughts are always related to an affective feeling that is indicating an unsolved problem. These ideas would correspond to preconscious representations (thing and word presentations) that are units used in working memory to help to create several options to satisfy pending needs.

The default mode network (DMN) may be another useful clinical resource to infer the contents of unresolved needs. It is activated when a person is in a resting state that does not recruit cognitive attention (Raichle et al., 2001) favoring internally focused tasks such as autobiographical memory retrieval, thinking about the future and mind wandering amongst others (Buckner et al., 2008). Carhart-Harris and Friston (2010) proposed that it suppresses prediction errors. The DMN may be at work while a person spontaneously is in a resting state. When patients are advised to retrieve some of their resting-state ideas, they tend to identify some of their pending needs and problems, for example, during prolonged sleep latencies due to insomnia.

(4) The last dimension uses the neurobiological correlates of affect as a resource to understand the subjective experience of a person. Panksepp’s lifelong work has left us with numerous documents in which he described key neuroanatomical and neurochemical correlates of the seven basic emotion systems (e.g., Panksepp, 1998, 2011; Panksepp and Biven, 2012). Understanding the neurochemical cascades and specific anatomical sites of these circuits provides further possibilities to integrate data and comprehend affects. Clinicians must observe that using this neurobiological knowledge allows only to infer what may be happening with the seven basic emotion systems. It cannot be affirmed with certainty that a given circuit is active or not without the appropriate testing. Again, this elaborates on the importance of relying on different dimensions of analysis to further underpin clinical hypotheses.

### Psychopathology Based on the Analysis of Affect

A topic that requires much work is that of symptoms and psychoanalytic diagnosis. It is classically accepted that there are three main diagnoses in psychoanalysis (e.g., Bergeret, 1974, 1975a; Kernberg, 1975; McWilliams, 2011), i.e., the neuroses, the psychoses, and narcissistic and borderline organizations. Each diagnosis requires a particular technique for different clinical objectives. Describing each of them in depth goes beyond the scope of this paper as they are topics that would require separate elaboration. Notwithstanding a general comprehension of common psychopathological issues is necessary to address clinical applications of neuropsychoanalytic knowledge.

As explained before, there is an adaptive reason why we must feel unpleasant affects. To try to avoid them, we must identify from the most basic level of consciousness, anoetic in Tulving’s classification of consciousness (Tulving, 1985), what problems require to be solved to survive. When instinctual behaviors, learned associations and cognitive thinking and planning have resulted unsuccessful for a prolonged period of time, a symptomatic formulation may arise as a result of using defenses as an attempt to diminish negative feelings. The way in which a defense reduces the intensity of a negative affect is by distorting reality; when facts are not fully perceived or acknowledged, then they should not keep the ability to produce unpleasurable feelings. However, affect is kept intact now just dissociated from its original stimulus or representation; thus, it looks for a new representation to associate with forming a symbolic symptom (Freud, 1894). This description delineates the formation of a neurotic symptom that can be interpreted. Freud proposed the existence of unconscious fantasies that led to intrapsychic conflict and scarcely worked on the topic of trauma after 1897, more common in narcissistic and borderline organizations. In these cases, patients suffer from intense separation distress due to narcissistic injuries related to abandonment, rejection, abuse, and/or ambivalent feelings from the primary objects (Flores Mosri, 2017, 2019a). This type of formulation excludes the intrapsychic conflict and instead is characterized by pervasive and persistent negative affects derived from the traumatic experience that recruits several structures, amongst others, the amygdala, a limbic structure that associates feelings of fear and/or anger with determined stimuli. What the amygdala learns is hardly forgotten, which can be explained evolutionarily as a means to predict future threatening situations. If the association is kept, the likelihood to quickly react in similar contexts is enhanced. Hence, traumatic experiences cannot be forgotten and provoke chronic unpleasant feelings that people naturally would like to dismiss. Traumatic experiences are a good instance to assess if an affect is regulated or not. It may be easy to assume that persistent negative affects are dysregulated. However, we must keep in mind that negative affects indicate an unsatisfied need or an unresolved problem. Thus, the stimuli that trigger negative affects should be identified and comprehended to evaluate whether there is a regulated emotional reaction to a stimulus or not. When the reaction is appropriate, affect is regulated despite its unpleasurable features. Trauma is a secondary-process emotional memory that must be dealt with through new learning that helps to formulate alternative resolution options to experiences that cannot be solved or deleted from memory. Yet, the original association tends to be predominant due to its predictive and adaptive character. Solms (2017) has explained that this type of implicit memory cannot be reconsolidated, i.e., it cannot become cognitively conscious and have access to the resources of working memory. Implicit memories are emotional and behavioral patterns and not representations. Neuropsychoanalytic knowledge then informs the clinician about what can be expected and what will not be achieved as therapeutic goals.

Then comes a frequent trauma derived from prolonged separation distress (PANIC/GRIEF) present in many psychopathological formulations, mainly related to narcissistic and borderline disorders. As human beings we are mammals which means that we depend on other human beings to be able to survive. Mammalian species depend on a primary caregiver to satisfy their needs during early life. The latter recruits the need to predict to survive (Friston, 2010; Fotopoulou and Tsakiris, 2017). Infants are taught by their parents how to solve their problems by reliably being available, usually providing an environment of certainty that results in basic trust (Erikson, 1950). The infant then learns how to avoid prediction errors and minimize free energy, which in turn means positive affective feelings. When early life has been characterized by the opposite, namely, uncertainty, the person is unable to learn how to build efficient predictions. The defense mechanisms used against negative affects impede the appropriate update that should follow a prediction error. This context favors enhanced feelings of uncertainty that relate to Panksepp’s PANIC/GRIEF system when the primary caregiver is not reliably available, to FEAR of traumatic experiences, and RAGE against the consequent excessive frustrations that such a context entails. These subjective feelings will not cease until the problems that they are indicating are solved. If that does not happen, a chronic negative feeling commands the person’s psychic life. It is then suggested to add to the analysis of affect the symptom formation. Symptoms are the best attempt to solve a problem or to satisfy needs; namely, they are an attempt to adapt and survive. When present, they express that the solution has not been found and that thus, the negative feelings that provoke them will endure. Solms (2017) refers to repression as an illegitimate or premature automatization of predictions that results in the chronic repetition of failed behavioral patterns. An alternative view of the latter refers to the simple unconscious learning of traumatic associations that are deeply rooted in structures that do not generate representations, such as the amygdala and the nucleus accumbens, generally related to reward (Berridge et al., 2009). As explained before, learned associations are not forgotten in order to predict threatening situations. If enhanced remembering of these contexts improves the possibility of surviving, then the negative affect must remain active. Overcoming trauma then entails accepting the defensive and adaptive objective of emotional memory.

In cases such as protracted separation distress, when the primary caregivers were not able to be predictable or when they were not able to provide the infant with a safe environment, chronic depressive feelings may arise (Marty, 1966; Bergeret, 1975b; Watt and Panksepp, 2009; Flores Mosri, 2019a). This context is useful to illustrate two instances. The first is that some needs and problems cannot be solved. If parents are unable to be reliable, there is not much that the infant can do to modify this situation. The unsatisfied need will persistently cause negative affect indications that promote various symptom formations. Hence, the second instance is that the psychotherapeutic process should include mourning for what cannot be solved. It implies the challenging task of accepting the frustration that derives from unsolved problems and generating alternative legitimate strategies to handle the existence of chronic negative feelings.

Thus, the idea that repetition compulsion is an expression of a death drive is challenged by neuropsychoanalytic knowledge. The reason why unsuccessful behavioral patterns are repeated relates to the involvement of procedural and emotional memory systems that recruit neuroanatomical structures that automatize the associations that they learn, for example the basal ganglia and the amygdala. Cognitive understanding of why one smokes does not stop the addictive behavior. This example calls for a top-down regulation of affective associations based on voluntarily inhibiting the repetition of inefficacious behavior patterns creating a conflict between spontaneous responses and the planned decision to try alternative solutions facilitated by working memory thinking. However, it should be mentioned that this type of top-down regulation means such a significant effort that many people fail on repeated attempts. Clinicians can easily interpret a death drive under these circumstances. No neurobiological evidence has been found to support Freud’s hypothesis of a death drive or instinct. All types of negative affects aim at driving the person toward a specific action that will solve problems. When a person repeatedly fails, frustration stops being a useful drive for resolutive actions. The result is a feeling of protracted RAGE (Flores Mosri, 2019a) that impacts the SEEKING system leading it to an eventual shutdown (Watt and Panksepp, 2009) that is felt as hopelessness. The clinician may notice then that it is not a death drive, but a depressive process that is at play. To sum up, symptoms do not intend to lead a person to their death; symptoms always attempt a solution of problems with the objective of surviving.

## Discussion: A Neuropsychoanalytic Psychotherapy Revolves Around Affect

From the proposed applications of neuropsychoanalysis to clinical work, it can be said that a neuropsychoanalytically informed psychotherapy takes affect as its central element. The affective nature of consciousness challenges what had been considered the main task of psychoanalysis. Solms (in press a) has argued that drives are conscious as so is the id (Solms, 2013). The neurobiological data supporting these novel notions demand an update of psychoanalytic concepts and technique. The reason why people consult is because they feel bad. They come to a psychotherapeutic process because they intend to stop feeling negative affects. A thorough analysis of emotions is then required. Psychoanalytic techniques have mainly worked without the valuable contributions of other fields, which render it vulnerable to errors at the cost of the patient’s suffering. Affect is a topic that must be studied from different perspectives to be properly understood. The analysis here proposed considers distinct dimensions of affect that are inextricably associated. All topics in psychoanalytic theory relate to affects, e.g., defense, the unconscious, symptoms, dreams, object relations, transference, countertransference, the Oedipus complex, the mental apparatus, memory, drives and instincts. Hence, it is difficult to think of any topic that could possibly have more relevance to clinical applications.

Analyzing the different dimensions of affect allows the clinician to understand what the patient suffers from, which in turn enhances the possibility of designing a treatment specific for the case’s features. The clinician must keep in mind that every thought expressed in language is based on emotions. If a person has difficulties with the subjective experience of negative affects, it is most likely that their thoughts will present various forms of distortion that impede accurate reconstructions of the past. Thus, an emphasis is placed on the transference-countertransference quality of the therapeutic relationship in which the clinician not only listens to what the patient speaks, but also feels the environment of the session (Balint, 1968) to better understand the patient’s affective problems in order to find the best solution available that may improve the person’s subjective experience.

However, the concept of homeostasis should be taken with caution, as it is an ideal. If a person could solve all their needs and problems, there would be no drive at all. Organisms constantly renew their needs creating novel challenges that drive toward motivated behaviors guided by a pleasure principle that aims at survival. We then live to feel multiple frustrations derived from our prediction errors. A psychotherapeutic process aims at minimizing entropy, namely the risk of dispersion or death (Friston, 2010; Wright and Panksepp, 2012; Solms, 2019). To succeed, the best prediction possible is to take account of a constant uncertainty that cannot be anticipated. The more a mind is ready to accept a certain amount of variability, then it is realistic enough as to know that an expected amount of frustration is inevitable and requires the task of mourning for what cannot be while remaining creative enough to find the best solutions possible for its affective existence.

There may be some concerns about whether the present proposal is suitable for psychoanalytic treatments. That remains to be discussed further, however, it may contribute to what could eventually be called a neuropsychoanalytically informed treatment.

## Author Contributions

The author confirms being the sole contributor of this work and has approved it for publication.

## Conflict of Interest

The author declares that the research was conducted in the absence of any commercial or financial relationships that could be construed as a potential conflict of interest.

## Publisher’s Note

All claims expressed in this article are solely those of the authors and do not necessarily represent those of their affiliated organizations, or those of the publisher, the editors and the reviewers. Any product that may be evaluated in this article, or claim that may be made by its manufacturer, is not guaranteed or endorsed by the publisher.

## Footnotes

1 Negative affect in this paper refers to affects that feel bad while positive affect refers to affects that feel good.

2 HM was a patient who suffered from intractable epilepsy. He agreed to undergo a surgery to bilaterally remove medial structures of the temporal lobe, including the hippocampi. As a result of the procedure, HM presented with severe anterograde amnesia and could not establish new autobiographical memories. Remarkably other memory systems were intact, i.e., procedural and working memory.

3 The use of capital letters is to identify neural circuits, as opposed to the ordinary use of these same words.
